# Repeated infusion of mesenchymal stem cells maintain the condition to inhibit deteriorated motor function, leading to an extended lifespan in the SOD1G93A rat model of amyotrophic lateral sclerosis

**DOI:** 10.1186/s13041-021-00787-6

**Published:** 2021-05-07

**Authors:** Hirotoshi Magota, Masanori Sasaki, Yuko Kataoka-Sasaki, Shinichi Oka, Ryo Ukai, Ryo Kiyose, Rie Onodera, Jeffery D. Kocsis, Osamu Honmou

**Affiliations:** 1 Department of Neural Regenerative Medicine, Research Institute for Frontier Medicine, Sapporo Medical University School of Medicine, Sapporo, Hokkaido 060-8556 Japan; 2 Tominaga Hospital, Naniwa-ku, Osaka-shi, Osaka, 556-0017 Japan; 3 Department of Neurology, Yale University School of Medicine, New Haven, CT 06510 USA; 4 Center for Neuroscience and Regeneration Research, VA Connecticut Healthcare System, West Haven, CT 06516 USA

**Keywords:** Amyotrophic lateral sclerosis, Mesenchymal stem cells intravenous, Multiple doses, Quality of life

## Abstract

Amyotrophic lateral sclerosis (ALS) is a neurodegenerative fatal disorder in which motor neurons within the brain and spinal cord degenerate. A single infusion of mesenchymal stem cells (MSCs) delays disease progression by protecting motor neurons and restoring the blood-spinal cord barrier in the SOD1G93A transgenic ALS rat model. However, the therapeutic effect of a single infusion of MSCs is transient and does not block disease progression. In this study, we demonstrated that repeated administration of MSCs (weekly, four times) increased the survival period, protected motor functions, and reduced deterioration of locomotor activity compared to a single infusion and vehicle infusion, after which rats displayed progressive deterioration of hind limb function. We also compared the days until gait ability was lost in rats and found that the repeated-infused group maintained gait ability compared to the single-infusion and vehicle-infusion groups. These results suggest that repeated administration of MSCs may prevent the deterioration of motor function and extend the lifespan in ALS.

**Supplementary Information:**

The online version contains supplementary material available at 10.1186/s13041-021-00787-6.

Amyotrophic lateral sclerosis (ALS) is a progressive neurodegenerative disorder in which motor neurons within the brain and spinal cord degenerate, leading to paralysis. Approximately 50% of patients with ALS die within 30 months of symptom onset, and approximately 20% of patients survive between 5 and 10 years after symptom onset [1], emphasizing the need for new therapies. Recent studies have reported that disruption of the blood-spinal cord barrier (BSCB) may contribute to the degeneration of large motor neurons in patients with ALS [2, 3] and animal models of ALS [4–7], regardless of genetic mutations. Single intravenous administration of mesenchymal stem cells (MSCs) has shown therapeutic effects for ALS [8] as well as cerebral ischemia [9, 10], spinal cord injury [11], and cerebral small vessel disease [12] via multiple and orchestrated mechanisms, including neuroprotection and restoration of the BSCB. We have previously reported that a single infusion of MSCs delays disease progression compared to vehicle infusion through the protection of motor neurons and restoration of the BSCB in the SOD1G93A transgenic ALS rat model [8]. However, the therapeutic effect of a single infusion of MSCs is transient, and disease progression continues as is characteristic of ALS. In this study, we tested whether repeated administration of MSCs extends the survival period and maintains better motor function compared to a single infusion of MSCs and vehicle in SOD1G93A rats.

When the Basso, Beattie, and Bresnahan (BBB) scoring scale [13] score became lower than or equal to 16 (normal locomotion 21, no movement 0), we started to infuse MSCs (MSC-1 group: single-infusion group) or vehicle (control group, no cells). At 7, 14, and 21 days after the first infusion, the repeated-infusion group (MSC-4 group: four times) was infused with MSCs, while the MSC-1 and control groups were infused with vehicle (fresh DMEM, without cells). The BBB scoring system was used to evaluate hind limb motor function by open-field locomotor activity, as described previously [14]. The test was performed twice a week from the age of 12 weeks to the endpoint (when rats no longer exhibited reflexes allowing them to right themselves within 30 s) (Fig. 1a) [14].

The median survival in the MSC-1 group (51 ± 0.2 days) was prolonged by 16.5 days compared with the control group (34.5 ± 0.2 days). The median survival of the MSC-4 group (77 ± 0.2 days) was 26 days longer than that of the MSC-1 group (p < 0.05) and 42.5 days longer than that of the control group (p < 0.05) (Fig. 1b). Thus, repeated infusion of MSCs significantly extended the lifespan of this rat ALS model. The number of animals in each group is presented in Additional file 1: Table S1.

In terms of serial changes in motor function evaluated via the BBB, we performed two types of analyses. One excluded rats whose BBB scores reached the endpoint (BBB = 0) (Fig. 1c) and the other included these rats (Fig. 1d). Figure 1c, where the animals whose BBB score reached the end-point (BBB = 0) were excluded, shows that scores on the BBB scale in the MSC-1 group were significantly higher than those of the control group at day 3 (16.6 ± 0.4 vs. 13.4 ± 0.3, p < 0.01, Mann–Whitney U test, p < 0.01), day 7 (14.6 ± 0.6 vs. 12.2 ± 0.6 p < 0.01), and day 28 (11.8 ± 0.7 vs. 6.2 ± 2.1, ANOVA and Tukey–Kramer’s post-hoc test, p < 0.05). Furthermore, the BBB score of the MSC-4 group was significantly higher than that of the control group at day 14 (14.9 ± 0.61 vs. 9.7 ± 1.28, ANOVA and Tukey–Kramer’s post-hoc test, p < 0.05), day 21 (14.6 ± 0.4 vs. 8.3 ± 1.7, p < 0.05), and day 28 (13.5 ± 0.7 vs. 6.2 ± 2.0, p < 0.01). The number of animals in each group is presented in Additional file 1: Table S2.

In Fig. 1d, where the animals whose BBB scores reached end-point (BBB = 0) were included in the analysis (n = 6/group), the BBB scores of the MSC-1 group are significantly higher than those of the control group at day 3 (16.6 ± 0.4 vs. 13.4 ± 0.3, Mann–Whitney U test, p < 0.01) and day 7 (14.6 ± 0.6 vs. 12.2 ± 0.6, p < 0.01). BBB scores of the MSC-4 group (n = 6) were significantly higher than of the control group (n = 6) on days 14 and 21 (14.6 ± 0.4 vs. 6.9 ± 2.0, ANOVA and Tukey–Kramer’s post-hoc test, p < 0.05), day 28 (13.5 ± 0.7 vs. 4.7 ± 2.2, p < 0.01), day 35 (11.8 ± 1.2 vs. 3.7 ± 2.0, p < 0.01), and day 42 (10.5 ± 1.3 vs. 1.8 ± 1.8, p < 0.01) after infusion (Fig. 1d). These results indicate that repeated infusion of MSCs reduces the deterioration of motor deficits in ALS.

We also compared the number of days required to reach the BBB scores to 7 from 15. The MSC-4 group reached a BBB score of 7 at 60 ± 9.5 days, the control group at 25 ± 5.8 days (p < 0.01), and the MSC-1 group at 41 ± 6.2 days (Fig. 1e). Since gait abilities are lost at a BBB score of 7, the period with preserved gait ability was prolonged in the MSC-4 group [13]. We also measured the body weight and found that the MSC-4 group maintained body weight during the study period. However, the MSC-1 and control groups lost body weight, although the body weight of rats in the MSC-1 group tended to be higher than that of rats in the control group. Note that body weights in the MSC-4 group were higher than in the control group at 28 days (292 ± 8.7 g vs 238 ± 12.1 g, p < 0.05) and 35 days (283 ± 6.7 g vs 227 ± 13.6 g, p < 0.05) (Fig. 1f).

Collectively, these results suggest that repeated infusion of mesenchymal stem cells may help reduce the deterioration of motor function in ALS, leading to a prolonged life expectancy, while a single infusion of MSCs delays disease progression to a lesser degree [8].

The therapeutic mechanisms of these changes are not elucidated in the current study. Previous studies with experimental animal models suggest multimodal and orchestrated mechanisms of infused MSCs, including secretion of neurotrophic factors that provide neuroprotection and restoration of the BSCB [15]. We have shown that both protection of motor neurons and restoration of the BSCB might be key mechanisms of a single infusion of MSCs in ALS rats [8]. At this point, we can only hypothesize that the sustainable improvement in behavioral function with repeated MSC infusion was due to a mixture of the above therapeutic mechanisms.

Future studies should be performed to clarify the operative mechanism to exert greater functional improvement in the repeated infusion of MSCs providing greater therapeutic efficacy in a rat model of ALS.

Prolonged life expectancy and reduced deterioration of motor function with an extended period during which rats can walk may lead to an improved quality of life. We stress that repeated infusion of MSCs before severe deterioration of ALS is important. Thus, repeated infusions of MSCs are a potentially promising therapeutic option for ALS that should be explored.

**Fig. 1:**
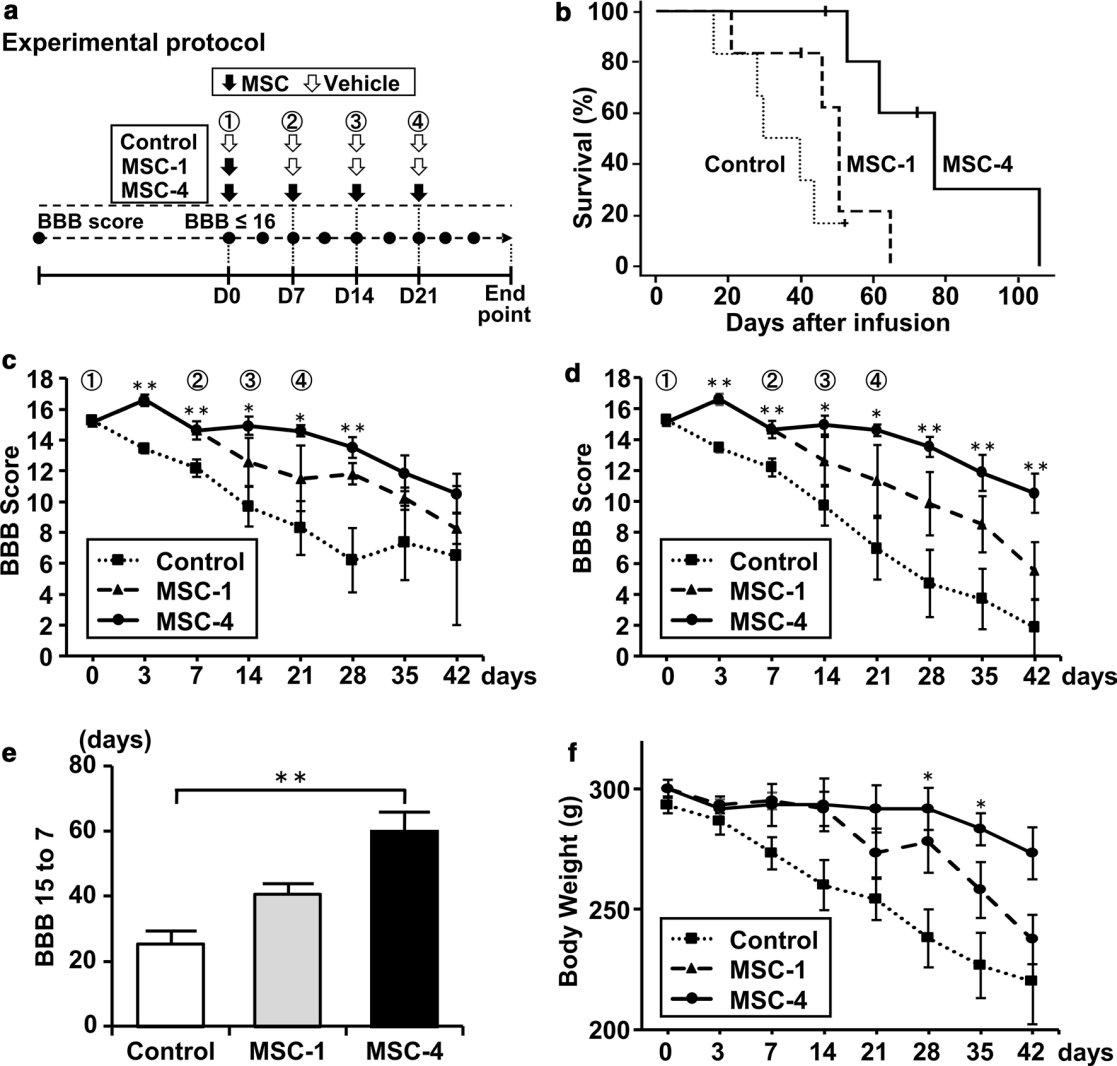
**a** Experimental protocol. **b** Kaplan–Meier estimates of mean survival of ALS rats. The MSC-4 group showed significantly prolonged survival using the log-rank test and Holm post-hoc test. Log-rank, p = 0.005. The number of surviving rats at each timer point are shown in Additional file 1: Table S1. **c** Basso-Beattie-Bresnahan (BBB) scores, excluding the rats that reached the endpoint (BBB score 0). The number of surviving rats at each timer point are shown in Additional file 1: Table S2. **d** BBB scores including the rats that reached the endpoint (BBB score 0) as zero. Arrows indicate the points of infusion. (n = 6/groups). **e** Required days to reach a BBB score of 7 (from BBB score 15). (n = 6/groups). **f** Body weight of all surviving animals. (n = 6/groups). *p < 0.05, **p < 0.01. Data are presented as mean ± SEM. *MSCs* mesenchymal stem cells, *BBB* Basso, Beattie, and Bresnahan, *SEM* standard error of the mean

## Supplementary Information

**Additional file 1: Table S1.** The number of surviving rats at each time point. **Table S2.** The number of surviving rats at each time point. We excluded rats whose BBB score was 0.

## Data Availability

The datasets used and analyzed in the current study are available from the corresponding author upon reasonable request.

## Abbreviations

ALS: Amyotrophic lateral sclerosis
DMEM: Dulbecco's modified Eagle's medium
MSCs: Mesenchymal stem cells
BSCB: Blood-spinal cord barrier
BBB: Basso, Beattie, and Bresnahan
