# Supplementary material for: Repeated infusion of mesenchymal stem cells maintain the condition to inhibit deteriorated motor function, leading to an extended lifespan in the SOD1G93A rat model of amyotrophic lateral sclerosis
Source: Mol Brain. 2021 May 7;14:76. doi: 10.1186/s13041-021-00787-6 (PMC8103621; doi:10.1186/s13041-021-00787-6)
Supplement: Supplementary file 1 — Additional file 1: Table S1. The number of surviving rats at each time point. Table S2. The number of surviving rats at each time point. We excluded rats whose BBB score was 0. [file 13041_2021_787_MOESM1_ESM.docx]

*Micro report*

**Repeated infusion of mesenchymal stem cells maintain the condition to inhibit deteriorated motor function, leading to an extended lifespan in the SOD1G93A rat model of amyotrophic lateral sclerosis**

Hirotoshi Magota ^1, 2^, Masanori Sasaki ^1, 3, 4*^, Yuko Kataoka-Sasaki ^1^, Shinichi Oka ^1^, Ryo Ukai ^1^, Ryo Kiyose ^1, 2^, Rie Onodera ^1^, Jeffery D. Kocsis ^3, 4^, Osamu Honmou ^1, 3, 4^

**Materials and Methods**

**Animals**

As an animal model of amyotrophic lateral sclerosis (ALS), female hemizygous (tg/wt) (n = 18) NTac:SD-Tg (SOD1G93A)L26H rats aged 5–12 weeks were purchased from Taconic Bioscience (Rensselaer, NY). All evaluations were performed by researchers who were blinded to the experimental conditions. Rats were considered end-stage (endpoint) when they no longer exhibited reflexes that allowed them to right themselves within 30 s (1).

**Preparation of mesenchymal stem cells from rat bone marrow**

Mesenchymal stem cells **(**MSC) culture preparation was based on a previous study (2). Briefly, bone marrow was obtained from the femoral bones of adult (6–8 weeks old) rats (n= 4), diluted in Dulbecco's modified Eagle's medium (DMEM) (Millipore Sigma, St. Louis, MO, USA) to a volume of 15 mL, supplemented with 10 % heat-inactivated fetal bovine serum (Thermo Fisher Scientific Inc., Waltham, MA, USA), 2 mM l-glutamine (Millipore Sigma), 100 U/mL penicillin, and 0.1 mg/mL streptomycin (Thermo Fisher Scientific Inc.), and was then incubated for three days at 37 °C in a humidified atmosphere containing 5 % CO_2_. When cultures were almost confluent, the adherent cells were detached with a trypsin-EDTA solution (Millipore Sigma) and cultured in 1 × 10^4^ cells/mL medium. MSCs were used after three passages. A previous phenotypic analysis of the surface antigens revealed a cluster of differentiation (CD) 45-, CD73+, CD90+, and CD106- on MSCs (3).

**Experimental procedure**

The ALS rats were randomized into three groups and anesthetized with an intraperitoneal (IP) injection of ketamine (75 mg/kg) and xylazine (10 mg/kg). The left femoral vein was carefully exposed and cannulated using a PE-10 tube connected to a 1-mL syringe. In the MSC-infused groups, rats were injected intravenously with MSCs (1.0 × 10^6^ cells each) in 1 mL total fluid volume (fresh DMEM) through the femoral vein. We used leftover cells to confirm the high cell viability (> 99 %) with 0.4 % trypan blue immediately after the procedure (2). All rats, including the control group, were injected daily with cyclosporine A (10 mg/kg, IP) (2). Since we focused on changes in hind limb function following MSC infusion, animals that displayed motor deficits in their forelimbs were not included.

**Behavioral testing**

We used the Basso, Beattie, and Bresnahan (BBB) scoring system (4) to evaluate the behavioral function in ALS rats as described previously (1). Rats were considered end-stage (endpoint) when they no longer exhibited reflexes that allowed them to right themselves within 30 s. Briefly, each animal was allowed to walk around the floor, while we evaluated hind limb and forelimb movements for approximately three to five minutes. Each hind limb score was based on a 21-point scoring scale from no movement (0) to normal locomotion (21). Scoring considers paw rotation, toe clearance, weight support, the frequency of each, and the amount of movement occurring from each joint (1). ALS rats were included in this study when the BBB behavioral scoring scale score was < 16. At a BBB score of 16, rats showed consistent plantar stepping and consistent forelimb-hind limb coordination during gait. Furthermore, toe clearance frequently occurs during limb forward movement, and the predominant paw position is parallel at the initial contact and rotated at liftoff. Open-field locomotor activity was assessed using the BBB score twice a week from 12 weeks of age to the endpoint after MSC or vehicle infusion.

**Statistical analysis**

All statistical analyses were performed using EZR (version.1.52; Saitama Medical Center, Jichi Medical University, Saitama, Japan) (5), which is a graphical user interface for R (The R Foundation for Statistical Computing, Vienna, Austria) and is a modified version of R commander designed to add statistical functions frequently used in biostatistics. Survival statistics were analyzed using the Kaplan-Meier method with EZR software (version.1.52; Saitama Medical Center, Jichi Medical University, Saitama, Japan) (5). Statistical analysis of survival was performed using the Kaplan-Meier method with the log-rank test and Bonferroni-Holm correction. For multiple comparisons, we used one-way analysis of variance followed by the Tukey-Kramer post hoc test. Data are expressed as mean ± standard error of the mean. Differences were considered statistically significant if *p < 0.05 or **p < 0.01.

**Additional Tables**

Table S1. The number of surviving rats at each time point (Figure 1B).

| **Day** | **0** | **10** | **20** | **30** | **40** | **50** | **60** | **70** | **80** | **90** | **100** |
| --- | --- | --- | --- | --- | --- | --- | --- | --- | --- | --- | --- |
| Control (n) | 6 | 6 | 5 | 3 | 3 | 1 | 0 | 0 | 0 | 0 | 0 |
| MSC-1 (n) | 6 | 6 | 6 | 5 | 5 | 3 | 1 | 0 | 0 | 0 | 0 |
| MSC-4 (n) | 6 | 6 | 6 | 6 | 6 | 5 | 4 | 3 | 1 | 1 | 1 |

Table S2. The number of surviving rats at each time point. We excluded rats whose BBB score was 0 (Figure 1C).

| **Day** | **0** | **3** | **7** |  | **14** | **21** | **28** | **35** | **42** |
| --- | --- | --- | --- | --- | --- | --- | --- | --- | --- |
| Control (n) | 6 | 6 | 6 |  | 6 | 5 | 5 | 3 | 2 |
| MSC-1 (n) | 6 | 6 | 6 |  | 6 | 6 | 5 | 5 | 4 |
| MSC-4 (n) | 6 | 6 | 6 |  | 6 | 6 | 6 | 6 | 6 |

**References**

1. Suzuki M, McHugh J, Tork C, Shelley B, Klein SM, Aebischer P, et al. GDNF secreting human neural progenitor cells protect dying motor neurons, but not their projection to muscle, in a rat model of familial ALS. PLoS One. 2007;2(8):e689.

2. Magota H, Sasaki M, Kataoka-Sasaki Y, Oka S, Ukai R, Kiyose R, et al. Intravenous infusion of mesenchymal stem cells delays disease progression in the SOD1G93A transgenic amyotrophic lateral sclerosis rat model. Brain Res. 2021:147296.

3. Kim S, Honmou O, Kato K, Nonaka T, Houkin K, Hamada H, et al. Neural differentiation potential of peripheral blood- and bone-marrow-derived precursor cells. Brain Res. 2006;1123(1):27-33.

4. Basso DM, Beattie MS, Bresnahan JC. A sensitive and reliable locomotor rating scale for open field testing in rats. J Neurotrauma. 1995;12(1):1-21.

5. Kanda Y. Investigation of the freely available easy-to-use software 'EZR' for medical statistics. Bone Marrow Transplant. 2013;48(3):452-8.
